# Association between pravastatin use and short-term mortality in ICU patients with sepsis: a retrospective propensity score-matched cohort study

**DOI:** 10.3389/fcimb.2026.1814760

**Published:** 2026-07-03

**Authors:** Bo Xie, Bo Zhou, Hong Teng, Jin-Qi Li, Jing Wang, Xiao-Jiao Cui

**Affiliations:** 1 Department of Cardiology, Chengdu Integrated TCM & Western Medicine Hospital, Chengdu, China; 2 Department of Pulmonary and Critical Care Medicine, Sichuan Provincial People’s Hospital, School of Medicine, University of Electronic Science and Technology of China, Chengdu, China; 3 Department of Pharmacy, Personalized Drug Therapy Key Laboratory of Sichuan Province, Sichuan Academy of Medical Sciences & Sichuan Provincial People’s Hospital, School of Medicine, University of Electronic Science and Technology of China, Chengdu, China; 4 Department of Pharmacy, Personalized Drug Research and Therapy Key Laboratory of Sichuan Province, Sichuan Provincial People’s Hospital, School of Medicine, University of Electronic Science and Technology of China, Chengdu, China

**Keywords:** intensive care unit, mortality, pravastatin, propensity score matching, sepsis

## Abstract

**Background:**

Sepsis is a life-threatening condition with persistently high mortality in the ICU, highlighting an urgent need for effective pharmacological interventions. Statins have been proposed as potential adjunctive therapies owing to their pleiotropic properties, but evidence specifically supporting the use of pravastatin—a hydrophilic statin with distinct pharmacokinetics—in this critically ill population remains insufficient.

**Methods:**

This retrospective cohort study utilized data from the MIMIC-IV database (version 3.1). Adult patients with sepsis at their first ICU admission who received pravastatin within the first 24 hours of ICU admission were included, while those using other statins were excluded. Propensity score matching (PSM) was performed at a 1:1 ratio to balance baseline characteristics between the pravastatin and non–pravastatin groups. The primary outcome was 28–day all–cause mortality. Secondary outcomes included in–hospital mortality, in–ICU mortality, and the incidence of acute kidney injury (AKI) and continuous renal replacement therapy (CRRT). Cox regression models were employed, and pre-specified subgroup analyses were conducted. Sensitivity analyses, including an expanded Cox model, overlap–weighted regression, and E–value analysis, were performed to assess the robustness of the findings.

**Results:**

From 546,028 admissions, 25,573 septic patients were identified. After PSM, 1,106 patients (553 per group) were included. The 28–day mortality rate was 11.6% (64/553) in the pravastatin group vs. 17.2% (95/553) in the non–pravastatin group. In the overall matched cohort, pravastatin use was associated with significantly lower 28–day mortality in both unadjusted (HR, 0.66; 95% CI, 0.48–0.90; P = 0.009) and fully adjusted models (HR, 0.69; 95% CI, 0.50–0.95; P = 0.025). Pravastatin was associated with reduced in-hospital mortality, while in–ICU mortality did not reach statistical significance after full adjustment. No significant differences were observed in AKI (79.6% vs. 81.7%; adjusted OR, 0.79; 95% CI, 0.57–1.10; P = 0.170) or CRRT use (4.0% vs. 5.8%; adjusted OR, 0.65; 95% CI, 0.32–1.30; P = 0.221). Subgroup analyses showed consistent associations across most strata, with no significant interactions (all P > 0.05). Timing analysis showed that in patients with pre-existing pravastatin use who continued treatment after ICU admission, continuation of pre-existing pravastatin was associated with reduced 28-day mortality (adjusted HR, 0.35; 95% CI, 0.17–0.73; P = 0.005). In patients with *de novo* initiation of pravastatin within 24 hours of ICU admission, no statistically significant association with 28-day mortality was observed after full adjustment (adjusted HR, 0.80; 95% CI, 0.57–1.13; P = 0.198). Sensitivity analyses yielded consistent results (expanded Cox: HR, 0.66; OW: HR, 0.70; E-value: 2.26 with lower bound 1.29).

**Conclusions:**

In this retrospective analysis of septic ICU patients, continuation of pre-existing pravastatin use after ICU admission was associated with lower 28-day and in-hospital mortality, without an increased risk of AKI or CRRT use. *De novo* pravastatin initiation within 24 hours of ICU admission was not associated with reduced 28-day mortality. These hypothesis–generating findings warrant confirmation in future prospective studies.

## Introduction

Sepsis, a dysregulated systemic inflammatory response syndrome triggered by infection, remains a leading cause of death in patients admitted to the intensive care unit (ICU), accounting for nearly 20% of global mortality (Seymour et al., 2016; Rudd et al., 2020; Evans et al., 2021). According to the Third International Consensus Definitions for Sepsis and Septic Shock, sepsis is defined as life-threatening organ dysfunction caused by a dysregulated host response to infection (Yang et al., 2020). Its pathophysiology involves a cascade of interconnected events, including dysregulated inflammatory response, endothelial dysfunction, coagulopathy, and immunosuppression (Joffre et al., 2020; Zhang and Ning, 2021; Liu et al., 2022; Girardis et al., 2024). Despite the widespread implementation of standardized strategies such as early fluid resuscitation, antimicrobial therapy, and organ support, the clinical outcomes of septic patients have not substantially improved. Currently, there is a lack of definitive and effective pharmacological interventions, and the high cost of treatment continues to impose a significant burden on global healthcare systems (Angus and van der Poll, 2013; Fleischmann et al., 2016; Lelubre and Vincent, 2018). Consequently, identifying adjuvant therapies with multi-pathway regulatory and pleiotropic effects has become a major research focus.

Statins, as inhibitors of 3-hydroxy-3-methylglutaryl-coenzyme A (HMG-CoA) reductase, are widely used cholesterol-lowering agents and a cornerstone of atherosclerotic cardiovascular disease prevention (Baigent et al., 2005; Arnett et al., 2019; Chou et al., 2022). Beyond lipid-lowering, accumulating evidence suggests that statins exert pleiotropic effects, including downregulation of pro-inflammatory cytokines, inhibition of macrophage proliferation, modulation of nitric oxide synthase balance, and improvement of endothelial function (Oesterle et al., 2017; Dehnavi et al., 2020; Koushki et al., 2021; Tajbakhsh et al., 2022; Yu and Liao, 2022; Kansal et al., 2023; Liu et al., 2023). These properties have prompted interest in repurposing statins for non-cardiovascular conditions such as dementia, cancer, asthma, and inflammatory lung diseases (Bradbury et al., 2018; Park et al., 2024; Park et al., 2024; Royall et al., 2024; Li et al., 2025; Westphal Filho et al., 2025), and support further exploration of their therapeutic potential in sepsis.

Accordingly, several studies have examined the association between statin use and outcomes in septic patients, with some large cohort studies reporting lower mortality among statin users (Terblanche et al., 2007; Lee et al., 2017; Li et al., 2024; Li et al., 2025), while others yielded neutral findings (Kruger et al., 2013; Wan et al., 2014). Importantly, most existing research has evaluated statins as a class, leaving the evidence regarding the association of specific statins with outcomes—particularly those with distinct pharmacokinetic properties—insufficiently explored. Pravastatin, a hydrophilic statin primarily cleared by the liver via active transport, differs from lipophilic statins in its metabolism and tissue distribution (Ford et al., 2016; Hague et al., 2016; Hodkinson et al., 2022). Preclinical studies suggest that pravastatin possesses anti-inflammatory, antioxidant, and endothelial-protective properties (Tong et al., 2022; Toghi et al., 2023), warranting further investigation in the context of sepsis. Given that large randomized trials have not shown improved survival with lipophilic statins (e.g., atorvastatin and simvastatin) in sepsis or acute respiratory distress syndrome (ARDS) (Kruger et al., 2013; Wan et al., 2014), and that clinical evidence specifically evaluating pravastatin in patients with severe sepsis remains scarce, pravastatin merits dedicated investigation.

Given the high prevalence of cardiovascular comorbidities and the substantial heterogeneity among patients with sepsis, understanding whether specific statins such as pravastatin are associated with improved outcomes may inform more targeted therapeutic approaches. To address this gap, we utilized a large-scale critical care database to evaluate the association between pravastatin use (including both continuation of pre-existing therapy and *de novo* initiation) and short-term mortality in critically ill patients with sepsis, providing real-world evidence to inform future research.

## Methods

### Data source

This retrospective cohort study utilized data from the Medical Information Mart for Intensive Care IV (MIMIC-IV, version 3.1) database. MIMIC-IV is a publicly accessible repository comprising de-identified health records of patients admitted to the Beth Israel Deaconess Medical Center from 2008 to 2019. The Institutional Review Board (IRB) at the institution granted ethical approval for the use of the database in research and waived the need for individual patient informed consent. Bo Xie and Xiao-Jiao Cui have passed the online training courses and exams (certification numbers: 74618521and 59921922). MIMIC-IV employs a modular data structure that enables linkage between different clinical modules and data modalities, which allowed us to ascertain out-of-hospital mortality status.

### Study population

**Figure 1:**
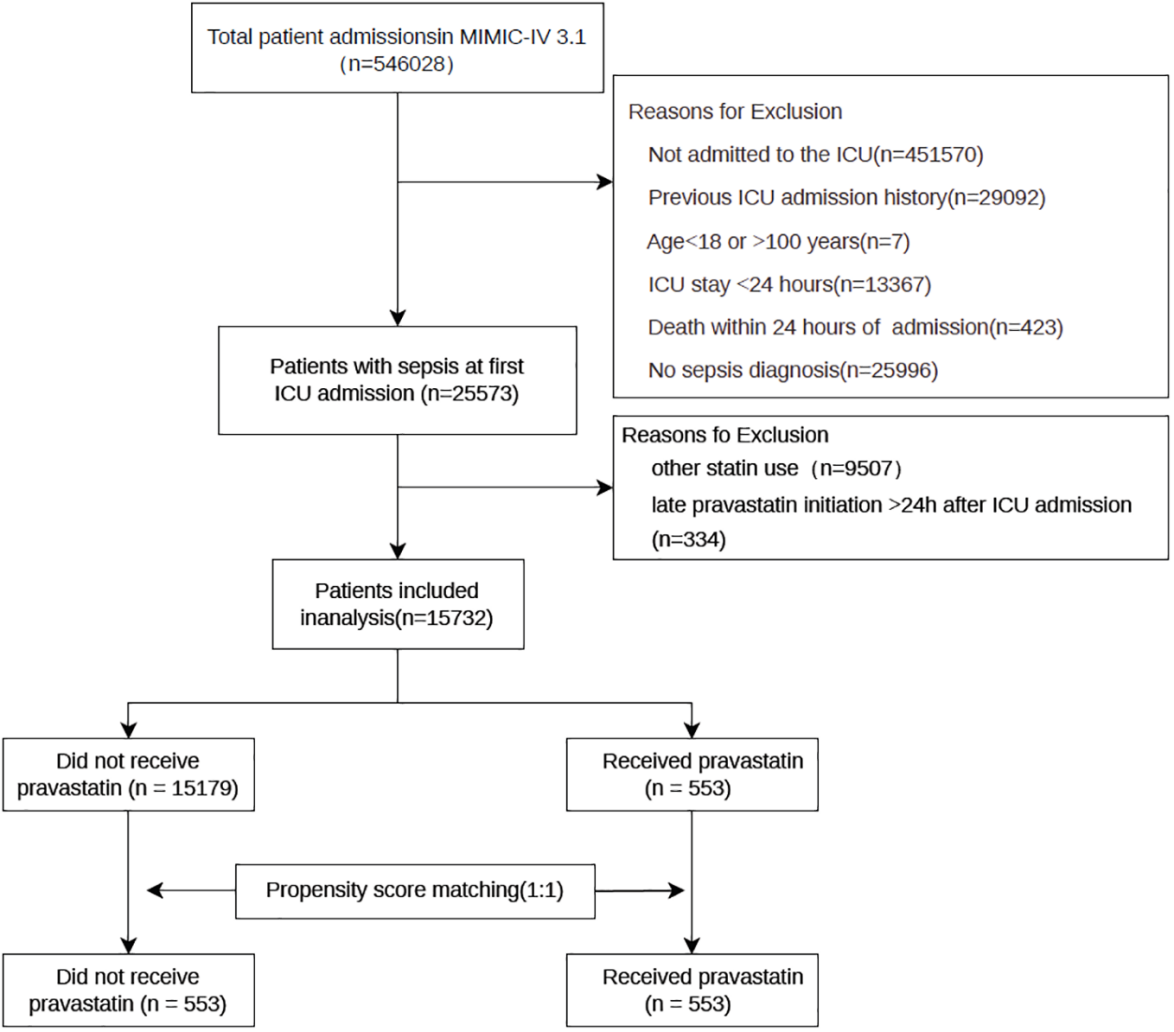
Flow chart of patient selection. MIMIC IV, Medical Information Mart for Intensive Care IV.

Sepsis was defined as a life-threatening organ dysfunction caused by a dysregulated inflammatory response to infection, as specified by the Third International Consensus Definitions for Sepsis and Septic Shock (Sepsis-3) in 2016 (Singer et al., 2016). This definition identifies suspected infection as the temporal co-occurrence of antibiotic administration and microbiological culture sampling, and organ dysfunction as a Sequential Organ Failure Assessment (SOFA) score of at least 2 within a window from 48 hours before to 24 hours after the suspected infection time. Selection criteria and the number of patients excluded at each step are shown in **Figure 1**. From a total of 546,028 patient admissions in the MIMIC-IV database (version 3.1), we excluded 451,570 admissions not involving ICU care, 29,092 patients with a prior ICU admission history, 7 patients aged below 18 or above 100 years, 13,367 patients with an ICU stay shorter than 24 hours, and 423 patients who died within 24 hours of admission to avoid immortal time bias. Additionally, 25,996 patients without a diagnosis of sepsis were excluded. This process identified 25,573 patients with sepsis at their first ICU admission.

We further excluded 9,507 patients who used other statins, and 334 patients who received pravastatin after the first 24 hours of ICU admission. Consequently, 553 patients who received pravastatin within the first 24 hours (including both continuation of pre-existing therapy and *de novo* initiation) and 15,179 who did not were included in the analysis. Subsequently, we performed 1:1 propensity score matching and matched 553 patients in the pravastatin group with 553 patients in the non-pravastatin group. The final matched cohort therefore comprised 1,106 patients.

### Data extraction

All data were extracted from the MIMIC-IV (version 3.1) database using Structured Query Language (SQL). The following data were collected within the first 24 hours following ICU admission: demographics (age, sex, race, weight); vital signs (heart rate, respiratory rate, temperature, SpO_2_, MAP); severity scores (SOFA score, Charlson Comorbidity Index); laboratory parameters (WBC, Hb, PLT, pH, PaO_2_, PaCO_2_, lactate, AG, sodium, potassium, total serum calcium, chloride, glucose, total bilirubin, ALT, AST, BUN, creatinine, INR); comorbidities (hyperlipidemia, SCAD, MI, CHF, ischemic stroke, hypertension, T2DM, COPD, cirrhosis, CKD, malignancy); and treatments within 24 hours (MV, vasoactive agent use, antibiotic use, glucocorticoid [GC] use, aspirin [ASA] use, and beta-blocker [BB] use). For vital signs and laboratory parameters, the first recorded value within the 24-hour window was used. For the SOFA score, the worst value (highest score) within the first 24 hours of ICU admission was used to capture the most severe acute physiological derangement.

### Exposure and study endpoints

The exposure of interest was pravastatin use within the first 24 hours of ICU admission. Patients who received pravastatin after the first 24 hours were excluded to minimize immortal time bias. Pravastatin use was extracted from the prescriptions table. Patients who did not receive pravastatin at any time during their ICU stay constituted the non-exposed group. The primary outcome of this study was 28-day all-cause mortality. Secondary outcomes included in-hospital mortality, in-ICU mortality, the incidence of acute kidney injury (AKI), and the incidence of continuous renal replacement therapy (CRRT).

### Statistical analysis

Baseline characteristics were summarized using descriptive statistics. Continuous variables are presented as mean ± standard deviation (SD) or median (interquartile range [IQR]), based on their distribution (Habibzadeh, 2017). Categorical variables are expressed as frequencies and percentages. Group comparisons were performed using the Kruskal-Wallis test for non-normally distributed continuous variables and Pearson’s chi-square test for categorical variables, as appropriate. Variables with more than 30% missing data were excluded from subsequent imputation. For covariates with missing values below this threshold, multiple imputation was applied to handle the missing data (Sterne et al., 2009; Mackinnon, 2010). The proportion of missing data for each variable is provided in **Supplementary Table 1**. We performed propensity score matching (PSM) to balance baseline covariates between groups according to the recommendations in the literature (Lonjon et al., 2017). PSM was performed at a 1:1 ratio using a caliper width of 0.2 standard deviations of the logit of the propensity score. The propensity score model included the following baseline covariates measured within the first 24 hours of ICU admission: demographic characteristics (age, sex, race, weight); vital signs (heart rate, respiratory rate, temperature, SpO_2_, MAP); severity scores (SOFA score, Charlson Comorbidity Index); laboratory parameters (WBC, Hb, PLT, pH, PaO_2_, PaCO_2_, lactate, AG, sodium, potassium, total serum calcium, chloride, glucose, total bilirubin, ALT, AST, BUN, creatinine, INR); comorbidities (hyperlipidemia, SCAD, MI, CHF, ischemic stroke, hypertension, T2DM, COPD, cirrhosis, CKD, malignancy); and treatments within 24 hours (mechanical ventilation, vasoactive agent use, antibiotic use, GC use, ASA use, BB use). The overlap of propensity score distributions between the pravastatin and non-pravastatin groups was assessed graphically before and after matching. As shown in **Supplementary Figure 1** (specifically the ‘Matched Data’ panel), there was substantial overlap in propensity score distributions after 1:1 matching, indicating that the matched groups had comparable baseline characteristics and supporting the appropriateness of the matching procedure. The balance of variables before and after matching was assessed using the standardized mean difference (SMD); an SMD value of less than 0.10 was considered to indicate adequate balance (Austin, 2009). The overlap of propensity score distributions between the matched groups is illustrated in **Supplementary Figure 1**.

To identify factors associated with mortality, we first performed univariate analyses in the cohorts before and after PSM (**Supplementary Tables 2, 3**, respectively). Multicollinearity was assessed using the variance inflation factor (VIF), with a VIF < 10 indicating no substantial multicollinearity (**Supplementary Table 4**). Variables that achieved p < 0.05 in univariate analysis and had a VIF < 10 were then included as covariates in the subsequent multivariable Cox regression models. Three multivariate models were constructed: Cox proportional hazards model was used to estimate hazard ratios (HRs) with 95% confidence intervals (CIs) for 28-day all-cause mortality, while logistic regression models were applied to derive odds ratios (ORs) with 95% CIs for in-hospital mortality, in-ICU mortality, the incidence of AKI, and the incidence of CRRT. Cumulative incidence curves for 28-day all-cause mortality were generated using the Kaplan–Meier method and compared with the log-rank test. Subgroup analyses were further conducted within the propensity score-matched cohort based on age (<65 vs. ≥65 years), gender, race (White vs. Other vs. Unknown), and key comorbidities including hyperlipidemia, ischemic stroke, stable coronary artery disease (SCAD), congestive heart failure (CHF), hypertension, type 2 diabetes mellitus (T2DM), chronic kidney disease (CKD), chronic obstructive pulmonary disease (COPD), and malignancy.

Several sensitivity analyses were conducted to assess the robustness of the primary findings. First, an expanded Cox model was fitted in the propensity score-matched cohort that included all variables with a univariate P < 0.05, without excluding those with a variance inflation factor (VIF) > 10. This approach tested whether the exclusion of highly collinear covariates in the primary model influenced the effect estimates. Second, overlap-weighted (OW) Cox regression was applied to balance baseline covariates while retaining the full cohort, thereby avoiding sample size reduction inherent to propensity score matching. Third, an E-value analysis was performed to quantify the potential impact of unmeasured confounding on the association between pravastatin use and 28-day mortality. The E-value represents the minimum strength of association that an unmeasured confounder would need to have with both the exposure and the outcome to fully explain away the observed effect.

The analyses were performed using R software (version 4.2.1; The R Foundation), Free Statistics software (version 2.4), and IBM SPSS Statistics (version 27).

## Results

### Cohort characteristics

**Table 1** presents the baseline characteristics of the study population before and after PSM. Before matching, significant differences were observed between the non-pravastatin group (n = 15,179) and the pravastatin group (n = 553). Patients receiving pravastatin were notably older (73.56 ± 11.65 vs. 62.81 ± 17.79 years) and had a higher prevalence of cardiovascular and metabolic comorbidities, including hyperlipidemia (77.6% vs. 24.7%), SCAD (18.3% vs. 5.4%), CHF (34.2% vs. 21.1%), hypertension (52.3% vs. 37.4%), and T2DM (43.0% vs. 21.5%). They also had higher weight (86.14 ± 23.01 vs. 82.04 ± 24.73 kg) and higher CKD (25.0% vs. 14.9%), but lower heart rate (83.31 ± 16.49 vs. 93.10 ± 22.41 beats/min), and respiratory rate (17.92 ± 7.01 vs. 20.08 ± 6.62 breaths/min). The SOFA score was comparable between the two groups before matching (5.0 [3.0, 7.0] vs. 5.0 [3.0, 8.0]).

**Table 1:** Baseline characteristics before and after propensity score matching.

Variable	Before propensity score matching		SMD	After propensity score matching		
	Non-pravastatin group (n, 15179)	Pravastatin group (n, 553)		Non-pravastatin group (n, 553)	Pravastatin group (n, 553)	SMD
Demographic Characteristics						
Age, mean ± SD, years	62.81 ± 17.79	73.56 ± 11.65	0.715	73.56 ± 12.79	73.56 ± 11.65	<0.001
Female gender, n (%)	6757 (44.5)	228 (41.2)	0.066	230 (41.6)	228 (41.2)	0.007
Race, n (%)			0.141			0.067
White, n (%)	9556 (63.0)	379 (68.5)		376 (68.0)	379 (68.5)	
Other, n (%)	3318 (21.9)	114 (20.6)		106 (19.2)	114 (20.6)	
Unknown, n (%)	2305 (15.2)	60 (10.8)		71 (12.8)	60 (10.8)	
Weight, mean ± SD, kg	82.04 ± 24.73	86.14 ± 23.01	0.172	85.00 ± 23.39	86.14 ± 23.01	0.049
Vital Signs						
Heart Rate, mean ± SD, beats/min	93.10 ± 22.41	83.31 ± 16.49	0.498	83.75 ± 17.74	83.31 ± 16.49	0.026
Respiratory Rate, mean ± SD, breaths/min	20.08 ± 6.62	17.92 ± 7.01	0.317	18.06 ± 5.97	17.92 ± 7.01	0.021
Temperature, mean ± SD, °C	36.89 ± 0.72	36.79 ± 0.57	0.151	36.79 ± 0.64	36.79 ± 0.57	0.004
SpO2, mean ± SD, %	96.77 ± 4.26	97.52 ± 3.54	0.193	97.39 ± 3.72	97.52 ± 3.54	0.036
MAP, mean ± SD, mmHg	81.26 ± 18.65	78.20 ± 17.13	0.171	78.38 ± 16.44	78.20 ± 17.13	0.011
Clinical Scores						
SOFA score, median (Q1, Q3)	5.0 (3.0, 8.0)	5.0 (3.0, 7.0)	0.221	5.0 (3.0, 7.0)	5.0 (3.0, 7.0)	0.003
Charlson Comorbidity Index,median (Q1, Q3)	5.0 (2.0, 7.0)	6.0 (4.0, 8.0)	0.370	6.0 (4.0, 7.0)	6.0 (4.0, 8.0)	0.005
Laboratory Parameters						
WBC, median (Q1, Q3), ×10^9^/L	11.6 (7.9, 16.4)	10.9 (8.1, 15.6)	0.074	11.8 (8.2, 15.6)	10.9 (8.1, 15.6)	0.025
Hb, mean ± SD, g/dL	10.58 ± 2.29	10.15 ± 1.89	0.207	10.27 ± 2.13	10.15 ± 1.89	0.061
PLT, median (Q1, Q3), ×10^9^/L	182.0 (121.0, 256.0)	166.0 (125.0, 231.0)	0.170	174.0 (129.0, 230.0)	166.0 (125.0, 231.0)	0.061
pH, mean ± SD	7.36 ± 0.10	7.38 ± 0.08	0.240	7.38 ± 0.09	7.38 ± 0.08	0.042
PaO2, median (Q1, Q3), mmHg	67.0 (42.0, 101.0)	79.0 (48.0, 113.0)	0.137	80.0 (48.0, 114.0)	79.0 (48.0, 113.0)	0.026
PaCO2, mean ± SD, mmHg	42.18 ± 12.68	41.85 ± 10.25	0.028	42.19 ± 11.08	41.85 ± 10.25	0.031
Lactate, median (Q1, Q3), mmol/L	1.8 (1.2, 2.8)	1.8 (1.3, 2.7)	0.143	1.7 (1.2, 2.6)	1.8 (1.3, 2.7)	0.004
AG, mean ± SD, mmol/L	14.86 ± 4.52	13.71 ± 3.85	0.273	13.84 ± 3.94	13.71 ± 3.85	0.033
Sodium, mean ± SD, mmol/L	138.24 ± 5.91	138.73 ± 4.52	0.095	138.98 ± 4.98	138.73 ± 4.52	0.052
Potassium, mean ± SD, mmol/L	4.19 ± 0.78	4.24 ± 0.69	0.071	4.22 ± 0.69	4.24 ± 0.69	0.034
Total Serum Calcium, mean ± SD, mg/dL	8.18 ± 0.93	8.32 ± 0.73	0.174	8.38 ± 0.85	8.32 ± 0.73	0.079
Chloride, mean ± SD, mmol/L	104.15 ± 7.21	105.51 ± 5.83	0.209	105.57 ± 6.77	105.51 ± 5.83	0.009
Glucose, median (Q1, Q3), mg/dL	128.0 (105.0, 163.0)	127.0 (108.0, 156.0)	0.007	130.0 (109.0, 164.0)	127.0 (108.0, 156.0)	0.029
Total Bilirubin, median (Q1, Q3), mg/dL	0.7 (0.4, 1.7)	0.6 (0.4, 1.1)	0.351	0.6 (0.4, 1.1)	0.6 (0.4, 1.1)	0.053
ALT, median (Q1, Q3), U/L	31.0 (17.0, 71.0)	22.0 (14.0, 41.0)	0.118	25.0 (15.0, 50.0)	22.0 (14.0, 41.0)	0.013
AST, median (Q1, Q3), U/L	44.0 (25.0, 101.0)	35.0 (22.0, 65.0)	0.121	36.0 (23.0, 60.0)	35.0 (22.0, 65.0)	0.002
BUN, median (Q1, Q3), mg/dL	20.0 (13.0, 35.0)	20.0 (15.0, 33.0)	0.022	21.0 (15.0, 33.0)	20.0 (15.0, 33.0)	0.020
Creatinine, median (Q1, Q3), mg/dL	1.0 (0.7, 1.6)	1.0 (0.8, 1.5)	0.011	1.0 (0.8, 1.5)	1.0 (0.8, 1.5)	0.026
INR, median (Q1, Q3)	1.3 (1.1, 1.6)	1.3 (1.2, 1.5)	0.071	1.3 (1.2, 1.5)	1.3 (1.2, 1.5)	0.044
Comorbidities						
Hyperlipidemia, n (%)	3749 (24.7)	429 (77.6)	1.246	432 (78.1)	429 (77.6)	0.013
SCAD, n (%)	826 (5.4)	101 (18.3)	0.405	101 (18.3)	101 (18.3)	<0.001
MI, n (%)	501 (3.3)	26 (4.7)	0.072	23 (4.2)	26 (4.7)	0.026
CHF, n (%)	3207 (21.1)	189 (34.2)	0.295	191 (34.5)	189 (34.2)	0.008
Ischemic Stroke, n (%)	1244 (8.2)	67 (12.1)	0.130	66 (11.9)	67 (12.1)	0.006
Hypertension, n (%)	5680 (37.4)	289 (52.3)	0.302	301 (54.4)	289 (52.3)	0.044
T2DM, n (%)	3264 (21.5)	238 (43.0)	0.473	240 (43.4)	238 (43.0)	0.007
COPD, n (%)	1041 (6.9)	63 (11.4)	0.158	66 (11.9)	63 (11.4)	0.017
Cirrhosis, n (%)	2005 (13.2)	21 (3.8)	0.342	25 (4.5)	21 (3.8)	0.036
CKD, n (%)	2255 (14.9)	138 (25.0)	0.255	129 (23.3)	138 (25.0)	0.038
Malignancy, n (%)	2311 (15.2)	94 (17.0)	0.048	89 (16.1)	94 (17.0)	0.024
Treatments and Medications						
MV within 24h after ICU admission, n (%)	6612 (43.6)	304 (55.0)	0.230	297 (53.7)	304 (55.0)	0.025
Vasoactive Agent Use within 24h after ICU admission, n (%)	4192 (27.6)	132 (23.9)	0.086	145 (26.2)	132 (23.9)	0.054
Antibiotic Use within 24h after ICU admission, n (%)	13283 (87.5)	506 (91.5)	0.131	508 (91.9)	506 (91.5)	0.013
GC Use, n (%)	3826 (25.2)	111 (20.1)	0.123	104 (18.8)	111 (20.1)	0.032
ASA Use, n (%)	3628 (23.9)	378 (68.4)	0.996	387 (70.0)	378 (68.4)	0.035
BB Use, n (%)	6081 (40.1)	380 (68.7)	0.601	384 (69.4)	380 (68.7)	0.016

AG, Anion Gap; ALT, Alanine Aminotransferase; ASA, Acetylsalicylic Acid; AST, Aspartate Aminotransferase; BB, Beta-adrenergic Blocking Agent; BUN, Blood Urea Nitrogen; CHF, Congestive Heart Failure; CKD, Chronic Kidney Disease; COPD, Chronic Obstructive Pulmonary Disease; GC, Glucocorticoid; Hb, Hemoglobin; INR, International Normalized Ratio; MAP, Mean Arterial Pressure; MI, Myocardial Infarction; MV, Mechanical Ventilation; PaCO_2_, Arterial Partial Pressure of Carbon Dioxide; PaO_2_, Arterial Partial Pressure of Oxygen; PLT, Platelet; SCAD, Stable Coronary Artery Disease; SOFA, Sequential Organ Failure Assessment; SpO_2_, Peripheral Oxygen Saturation; T2DM, Type 2 Diabetes Mellitus; WBC, White Blood Cell.

After 1:1 propensity score matching, 553 patients were included in each group, achieving well-balanced baseline characteristics. The SMDs for all variables were below 0.1, indicating that the distribution of baseline variables was similar between the two groups. The covariate balance after PSM is visualized using a Love plot (**Supplementary Figure 2**).

## Primary outcome

### 28-day all-cause mortality

In the original cohort, the 28-day all-cause mortality was 3,483 (22.9%) in the non-pravastatin group (n=15,179) and 64 (11.6%) in the pravastatin group (n=553), with a significant between-group difference (p < 0.001; see **Supplementary Table 2**). In the matched cohort, the 28-day mortality remained significantly lower in the pravastatin group (64/553, 11.6%) compared with the non-pravastatin group (95/553, 17.2%; p = 0.009; see **Supplementary Table 3**).

Univariate analyses for the after PSM cohort are presented in **Supplementary Table 3**, and multivariable analyses are shown in **Table 2**. In the unadjusted model (Model I), pravastatin use within the first 24 hours of ICU admission was associated with a 34% lower risk of 28–day mortality risk (HR, 0.66; 95% CI, 0.48–0.90; P = 0.009). After adjusting for demographic and vital signs (Model II), the association remained significant (HR, 0.66; 95% CI, 0.48–0.91; P = 0.012). In the fully adjusted model (Model III), which additionally accounted for laboratory parameters, comorbidities, severity scores, and concomitant medications, the association was somewhat attenuated but remained statistically significant (HR, 0.69; 95% CI, 0.50–0.95; P = 0.025).

**Table 2:** Multivariable cox regression analysis for 28-day all-cause mortality.

Categories	Model I	P-value	Model II	P-value	Model III	P-value
	HR/OR (95% CI)		HR/OR (95% CI)		HR/OR (95% CI)	
Primary Outcome						
28-day mortality*						
PSM Cohort (Pravastatin vs. Non-Pravastatin)	0.66 (0.48~0.9)	0.009	0.66 (0.48~0.91)	0.012	0.69 (0.5~0.95)	0.025
Secondary outcomes						
In-hospital mortality※						
PSM Cohort (Pravastatin vs. Non-Pravastatin)	0.57 (0.39~0.82)	0.003	0.56 (0.38~0.83)	0.004	0.54 (0.35~0.82)	0.004
In-ICU mortality※						
PSM Cohort (Pravastatin vs. Non-Pravastatin)	0.63 (0.41~0.98)	0.039	0.63 (0.4~0.99)	0.044	0.65 (0.39~1.07)	0.092
Incidence of AKI※						
PSM Cohort (Pravastatin vs. Non-Pravastatin)	0.87 (0.65~1.17)	0.361	0.82 (0.6~1.12)	0.213	0.79 (0.57~1.1)	0.17
Incidence of CRRT※						
PSM Cohort (Pravastatin vs. Non-Pravastatin)	0.67 (0.39~1.18)	0.165	0.67 (0.38~1.19)	0.176	0.65 (0.32~1.3)	0.221

AKI, Acute Kidney Injury; CI, Confidence Interval; CRRT, Continuous Renal Replacement Therapy; HR, Hazard Ratio.

* HR with 95% CI was calculated using the Cox proportional hazards model.

^※^OR with 95% CI was calculated using the logistic regression model.

Model I: did not adjust any variables.

Model II: adjusted for age, gender, race, weight, MAP, heart rate, respiratory rate, SpO_2_, temperature.

Model III: adjusted for age, gender, race, weight, MAP, heart rate, respiratory rate, SpO_2_, temperature, WBC, PLT, Chloride, Glucose, pH, Pco2, Po2, INR, ALT, BUN, Creatinine, Hypertension, MI, CHF, COPD, Ischemic stroke, SOFA score, Vasoactive Agent Use within 24h after ICU admission, BB Use, GC Use.

**Figure 2:**
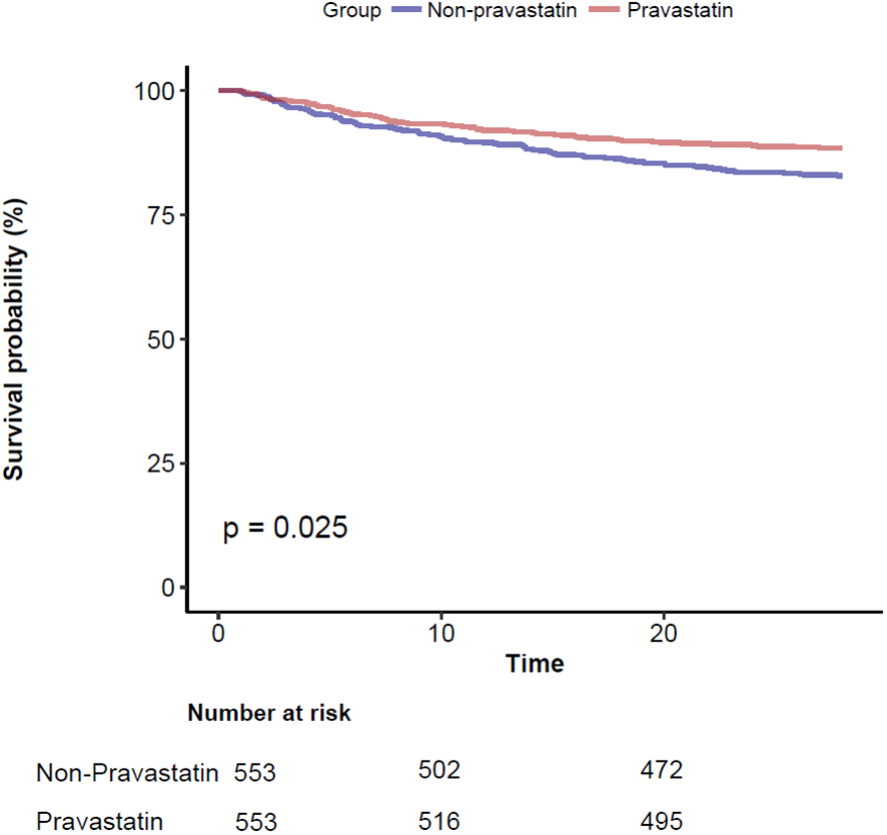
Kaplan-Meier curves for 28-day all-cause mortality according to pravastatin use in the matched cohort.

**Figure 2** presents the Kaplan–Meier curves for 28-day all-cause mortality stratified by pravastatin use within the first 24 hours of ICU admission. In the propensity score-matched cohort, patients who received pravastatin within the first 24 hours of ICU admission exhibited significantly higher survival probabilities compared with those who did not (log-rank P = 0.025).

### Subgroup analysis of pravastatin use timing and 28-day mortality

To further evaluate whether different pravastatin use patterns were associated with 28-day mortality, we compared three distinct groups: (1) Pre–Existing Pravastatin Group (patients with documented pre-admission pravastatin use who continued treatment within 24 hours after ICU admission); (2) New-Onset Pravastatin Group (patients without documented pre-admission pravastatin use who received their first dose within 24 hours after ICU admission); and (3) Non-Pravastatin Group (patients who never received pravastatin during their ICU stay). Pairwise comparisons were performed using Cox regression with Bonferroni correction for multiple comparisons (adjusted significance threshold α′ = 0.0167).

In the fully adjusted model (Model III), the Pre–Existing Pravastatin Group was associated with a significantly lower risk of 28–day mortality compared with the Non–Pravastatin Group (HR, 0.35; 95% CI, 0.17–0.73; unadjusted P = 0.005). In contrast, the direct comparison between the Pre–Existing Pravastatin Group and the New-Onset Pravastatin Group did not reach statistical significance after Bonferroni correction (HR, 0.39; 95% CI, 0.17–0.87; unadjusted P = 0.021), as the unadjusted P value exceeded the pre-specified threshold of 0.0167. The New-Onset Pravastatin Group did not show a statistically significant association with 28–day mortality compared with the Non–Pravastatin Group (HR, 0.80; 95% CI, 0.57–1.13; unadjusted P = 0.198) (**Table 3**).

**Table 3:** Comparison of 28-day mortality across different pravastatin use groups.

Categories	Model I	Unadjusted P value	Model II	Unadjusted P value	Model III	Unadjusted P value
	HR (95% CI)		HR (95% CI)		HR (95% CI)	
28-day mortality						
Pre–Existing Pravastatin Group vs. Non-Pravastatin Group	0.29 (0.14~0.59)	0.001	0.33 (0.16~0.68)	0.003	0.35 (0.17~0.73)	0.005
New-Onset Pravastatin Group vs. Non-Pravastatin Group	0.8 (0.58~1.12)	0.191	0.77 (0.55~1.07)	0.12	0.8 (0.57~1.13)	0.198
Pre–Existing Pravastatin Group vs. New-Onset Pravastatin Group	0.36 (0.17~0.76)	0.007	0.42 (0.2~0.9)	0.026	0.39 (0.17~0.87)	0.021

CI, Confidence Interval; HR, Hazard Ratio.

Model I: did not adjust any variables.

Model II: adjusted for age, gender, race, weight, MAP, heart rate, respiratory rate, SpO_2_, temperature.

Model III: adjusted for age, gender, race, weight, MAP, heart rate, respiratory rate, SpO_2_, temperature, WBC, PLT, Chloride, Glucose, pH, Pco2, Po2, INR, ALT, BUN, Creatinine, Hypertension, MI, CHF, COPD, Ischemic stroke, SOFA score, Vasoactive Agent Use within 24h after ICU admission, BB Use, GC Use.

Group Definitions: Pre–Existing Pravastatin Group = Patients with a documented history of pravastatin use before hospital admission, who continued pravastatin treatment within 24 hours after ICU admission; New-Onset Pravastatin Group = Patients with no documented history of pravastatin use before hospital admission, who received their first dose of pravastatin within 24 hours after ICU admission; Non-Pravastatin Group = Patients with no pravastatin after ICU admission.

Statistical Notes: The table presents unadjusted P values. Because three pairwise comparisons were performed for each model, statistical significance after Bonferroni correction was defined as a two-sided unadjusted P < 0.0167 (0.05/3). HRs compare the first group with the second group named in each row *.

## Secondary outcomes

### ICU mortality and in-hospital mortality

In the matched cohort, the ICU mortality rate was 6.5% (36/553) in the pravastatin group and 9.9% (55/553) in the non–pravastatin group. Logistic regression analysis showed that pravastatin use within the first 24 hours of ICU admission was associated with lower ICU mortality in the unadjusted model (OR, 0.63; 95% CI, 0.41–0.98; P = 0.039) and after adjustment for demographic and vital signs (Model II: OR, 0.63; 95% CI, 0.40–0.99; P = 0.044). However, in the fully adjusted model (Model III), the association was attenuated and no longer statistically significant (OR, 0.65; 95% CI, 0.39–1.07; P = 0.092) (**Table 2**).

The in-hospital mortality rate was 9.2% (51/553) in the pravastatin group and 15.2% (84/553) in the non-pravastatin group. Pravastatin use was associated with significantly lower in-hospital mortality across all three models: Model I (OR, 0.57; 95% CI, 0.39–0.82; P = 0.003), Model II (OR, 0.56; 95% CI, 0.38–0.83; P = 0.004), and Model III (OR, 0.54; 95% CI, 0.35–0.82; P = 0.004) (**Table 2**).

### Incidence of AKI and CRRT

The incidence of acute kidney injury (AKI) and the use of continuous renal replacement therapy (CRRT) did not differ significantly between the groups in the matched cohort. Specifically, AKI occurred in 79.6% (440/553) of the pravastatin group versus 81.7% (452/553) of the non–pravastatin group (Model III OR, 0.79; 95% CI, 0.57–1.10; P = 0.170). CRRT rates were 4.0% (22/553) in the pravastatin group and 5.8% (32/553) in the non–pravastatin group (Model III OR, 0.65; 95% CI, 0.32–1.30; P = 0.221) (**Table 2**).

### Subgroup analyses

**Figure 3:**
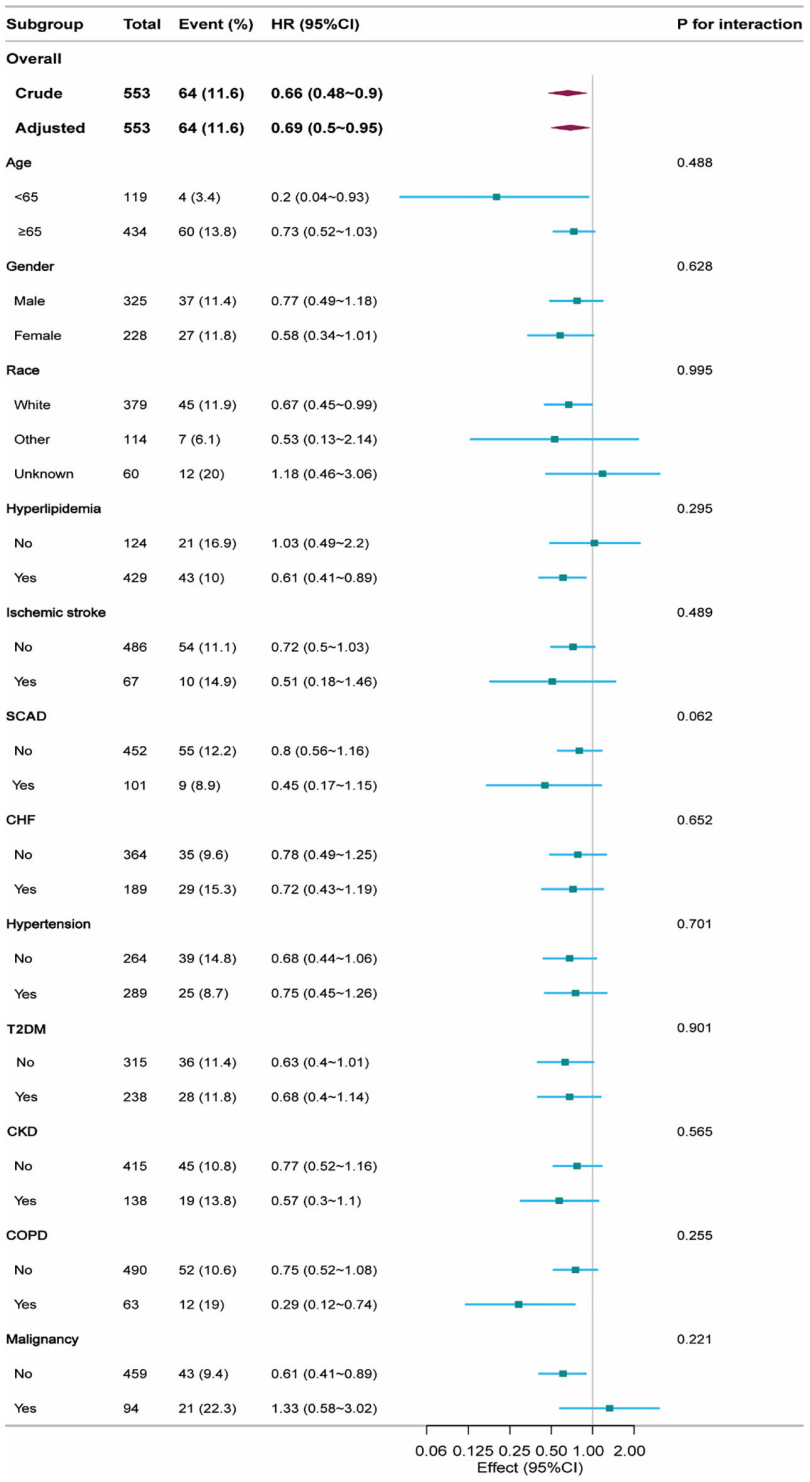
Subgroup analyses for 28-day all-cause mortality in the matched cohort.

Subgroup analyses were conducted to evaluate the consistency of the observed association across prespecified strata, including age (<65 vs. ≥65 years), gender, race, hyperlipidemia, ischemic stroke, SCAD, CHF, hypertension, T2DM, CKD, COPD, and malignancy. The observed association of pravastatin use with 28–day mortality was generally consistent across all subgroups, as no significant interactions were observed (all P for interaction > 0.05). Detailed results are presented in **Figure 3**.

### Sensitivity analyses

The results of the sensitivity analyses were consistent with the primary findings. An expanded Cox model that retained all candidate variables (without excluding those with VIF > 10) yielded an HR of 0.66 (95% CI, 0.47–0.93; P = 0.016). Overlap-weighted (OW) Cox regression produced an HR of 0.70 (95% CI, 0.54–0.91; P = 0.008). An E-value analysis for the primary HR of 0.69 gave a point-estimate E-value of 2.26 and a lower-bound E-value (for the 95% CI) of 1.29 (**Supplementary Table 5**).

## Discussion

This study evaluated the association of pravastatin in ICU patients with sepsis using data from the MIMIC-IV database and a rigorous PSM approach. The results showed an association that, compared with non-users, sepsis patients receiving pravastatin had a significantly lower 28-day all-cause mortality (HR, 0.69; 95% CI, 0.5–0.95; P = 0.025). This association remained consistent for in-hospital mortality. Meanwhile, a timing analysis revealed that this association was observed primarily in patients with pre-existing pravastatin use who continued treatment after ICU admission (HR, 0.35; P = 0.005), whereas *de novo* initiation did not show a statistically significant association after multiple comparison correction. Moreover, pravastatin use did not significantly increase the incidence of AKI or the requirement for CRRT. These findings provide hypothesis-generating observational evidence suggesting a potential adjunctive role of pravastatin.

Several recent observational studies and meta-analyses have reported an association between statin use and lower mortality in sepsis patients. Li et al (Li et al., 2025) and Li et al. (2024), in large-scale cohort studies, both found that statin therapy was associated with significantly lower mortality among sepsis patients in the ICU. Another study by Zhan et al. (2025) focusing on sepsis patients with AKI also indicated that early statin intervention was associated with higher 90-day survival rates. This study provides further support for the potential association between statin use and lower mortality in patients with sepsis, which is consistent with the trend of the above studies. The pathophysiological core of sepsis involves a dysregulated host response to infection, leading to excessive systemic inflammation, endothelial injury, microcirculatory dysfunction, and immunosuppression (Boomer et al., 2011; Joffre et al., 2020; Liu et al., 2022; Brandes-Leibovitz et al., 2024). Statins can downregulate the expression of pro-inflammatory cytokines (e.g., TNF-α, IL-6) by inhibiting signaling pathways such as nuclear factor κB (NF-κB) (Jain and Ridker, 2005; Zeiser, 2018). Additionally, statins enhance endothelial nitric oxide synthase (eNOS) activity, promote nitric oxide (NO) production, improve vasodilation and microcirculatory perfusion, and inhibit endothelial apoptosis and leukocyte adhesion (Broniarek et al., 2020; Liu et al., 2023; Chen et al., 2024). Immunologically, statins have been shown to modulate macrophage polarization, promote efferocytosis, and potentially ameliorate sepsis-induced immunosuppression (Tajbakhsh et al., 2022). These multi-target effects collectively form the theoretical basis for alleviating organ dysfunction and improving prognosis in sepsis.

However, findings from studies on statins in sepsis are not entirely consistent. Several randomized controlled trials (RCTs) evaluating statins such as atorvastatin and simvastatin in patients with severe sepsis, ARDS, or VAP have failed to show improved survival (van den Hoek et al., 2011; Kruger et al., 2013; McAuley et al., 2014; Wan et al., 2014). A critical appraisal of these trials reveals substantial differences in study design, patient populations, timing of statin use patterns (chronic pre-admission use versus *de novo* initiation in the ICU), baseline cardiovascular risk, and illness severity. Given the neutral results from these prior RCTs of other statins, our observational findings should be interpreted with caution. Whether pravastatin, with its distinct pharmacological properties (hydrophilicity and non-CYP metabolism), might yield different results remains an open question that warrants further investigation. This heterogeneity may be attributable to multiple factors, including study design, population differences, statin type, timing of administration (pre–hospital pretreatment vs. in–hospital *de novo* therapy), and dosage. One plausible contributor among these is the distinct pharmacological profile of pravastatin, although this interpretation remains speculative. Unlike lipophilic statins (e.g., simvastatin, atorvastatin), which are primarily metabolized via the cytochrome P450 (CYP) enzyme system, pravastatin is primarily taken up by hepatocytes via Organic Anion Transporting Polypeptide 1B1 (OATP1B1) and undergoes minimal CYP450-mediated metabolism (Elsby et al., 2012; Hirota et al., 2020; Cooper-DeHoff et al., 2022). In critical conditions such as sepsis, systemic inflammation, hepatic insufficiency, and complex polypharmacy often lead to unpredictable changes in CYP enzyme activity, thereby affecting the efficacy and safety of lipophilic statins (Carcillo et al., 2003; Zhang and Ning, 2021; Borges and Bento, 2024). Meanwhile, statins metabolized via CYP are more prone to drug interactions with other medications metabolized through the same enzyme system. Therefore, the use of pravastatin may be associated with lower mortality in critically ill patients. Future studies should directly compare different types of statins in septic populations to investigate whether pravastatin’s unique pharmacokinetic profile is associated with different clinical outcomes. Although patients in the pravastatin group were older and had more cardiovascular complications, no significant difference in the incidence of AKI or CRRT was observed between the two groups, which is consistent with previous reports (Tonelli et al., 2005; Gitomer et al., 2025).

A key finding from our timing analysis is that the observed association was present only in patients who continued pre-existing pravastatin use, whereas no statistically significant association was found for *de novo* initiation after ICU admission. This pattern warrants careful interpretation. One possibility is residual confounding, also known as healthy user bias: patients who are chronically prescribed statins may differ from non-users in unmeasured ways—such as better health awareness, more regular medical follow-up, or higher socioeconomic status—that could independently influence their outcomes. Alternatively, it is possible that the duration of pravastatin exposure prior to ICU admission (which we could not measure) plays a role, or that the pharmacological effects of pravastatin require longer exposure to manifest. However, given the observational nature of our data, we cannot distinguish between these possibilities. While we cannot rule out a true pharmacological effect, confounding remains a parsimonious explanation for the observed pattern.

### Study limitations

This study has several limitations. First, its retrospective observational design precludes definitive causal inference, and residual confounding from unmeasured variables cannot be completely ruled out. Although we adjusted for a broad range of available covariates—including severity scores (SOFA, Charlson Comorbidity Index), laboratory parameters, and key concomitant medications (antibiotics, glucocorticoids, aspirin, and beta-blockers)—we were unable to account for certain clinically relevant factors such as the focus and source of infection, specific microorganism, prior functional status, frailty, and quality of care, as these variables are not available in the MIMIC-IV database. Second, to address potential bias from variable exposure timing, we restricted the analysis to patients who received pravastatin within the first 24 hours of ICU admission and excluded those who received it after the first 24 hours. This approach helped ensure a clearly defined exposure window and reduce immortal time bias. Nevertheless, we acknowledge that while we were able to define in-hospital medication use timing precisely, information on pre-admission pravastatin use—including treatment duration and adherence prior to ICU admission—was not available. Third, the study evaluated only short-term mortality outcomes; the effects of pravastatin on long-term survival and quality of life remain to be investigated. Fourth, this study was not prospectively registered, which may raise concerns about selective reporting. Nevertheless, our analysis was guided by a pre–specified pharmacological hypothesis, and all analyses have been reported transparently. Fifth, the MIMIC-IV database does not provide reliable data on cause–specific mortality. Therefore, we were unable to determine whether the observed association was primarily driven by sepsis–related deaths or by other causes of death.

## Conclusions

In this retrospective analysis of septic ICU patients, continuation of pre-existing pravastatin use after ICU admission was associated with lower 28 day and in hospital mortality, without an increased risk of AKI or CRRT use. *De novo* pravastatin initiation within 24 hours of ICU admission was not associated with reduced 28-day mortality. These hypothesis–generating findings warrant confirmation in future prospective studies.

## Data Availability

The original contributions presented in the study are included in the article/**Supplementary Material**. Further inquiries can be directed to the corresponding authors.

## References

[B1] Angus D. C. van der Poll T. (2013). Severe sepsis and septic shock. N. Engl. J. Med. 369, 840–851. doi: 10.1056/NEJMra1208623 23984731

[B2] Arnett D. K. Blumenthal R. S. Albert M. A. Buroker A. B. Goldberger Z. D. Hahn E. J. . (2019). 2019 ACC/AHA guideline on the primary prevention of cardiovascular disease: a report of the american college of cardiology/american heart association task force on clinical practice guidelines. Circulation 140, e596–e646. doi: 10.1161/CIR.0000000000000678 30879355 PMC7734661

[B3] Austin P. C. (2009). Balance diagnostics for comparing the distribution of baseline covariates between treatment groups in propensity-score matched samples. Stat. Med. 28, 3083–3107. doi: 10.1002/sim.3697 19757444 PMC3472075

[B4] Baigent C. Keech A. Kearney P. M. Blackwell L. Buck G. Pollicino C. . (2005). Efficacy and safety of cholesterol-lowering treatment: prospective meta-analysis of data from 90,056 participants in 14 randomised trials of statins. Lancet Lond. Engl. 366, 1267–1278. doi: 10.1016/S0140-6736(05)67394-1 16214597

[B5] Boomer J. S. To K. Chang K. C. Takasu O. Osborne D. F. Walton A. H. . (2011). Immunosuppression in patients who die of sepsis and multiple organ failure. JAMA 306, 2594–2605. doi: 10.1001/jama.2011.1829 22187279 PMC3361243

[B6] Borges A. Bento L. (2024). Organ crosstalk and dysfunction in sepsis. Ann. Intensive Care 14, 147. doi: 10.1186/s13613-024-01377-0 39298039 PMC11413314

[B7] Bradbury P. Traini D. Ammit A. J. Young P. M. Ong H. X. (2018). Repurposing of statins via inhalation to treat lung inflammatory conditions. Adv. Drug Delivery Rev. 133, 93–106. doi: 10.1016/j.addr.2018.06.005 29890243

[B8] Brandes-Leibovitz R. Riza A. Yankovitz G. Pirvu A. Dorobantu S. Dragos A. . (2024). Sepsis pathogenesis and outcome are shaped by the balance between the transcriptional states of systemic inflammation and antimicrobial response. Cell Rep. Med. 5, 101829. doi: 10.1016/j.xcrm.2024.101829 39566468 PMC11604535

[B9] Broniarek I. Dominiak K. Galganski L. Jarmuszkiewicz W. (2020). The influence of statins on the aerobic metabolism of endothelial cells. Int. J. Mol. Sci. 21, 1485. doi: 10.3390/ijms21041485 32098258 PMC7073032

[B10] Carcillo J. A. Doughty L. Kofos D. Frye R. F. Kaplan S. S. Sasser H. . (2003). Cytochrome P450 mediated-drug metabolism is reduced in children with sepsis-induced multiple organ failure. Intensive Care Med. 29, 980–984. doi: 10.1007/s00134-003-1758-3 12698250

[B11] Chen W.-H. Chen C.-H. Hsu M.-C. Chang R.-W. Wang C.-H. Lee T.-S. (2024). Advances in the molecular mechanisms of statins in regulating endothelial nitric oxide bioavailability: interlocking biology between eNOS activity and L-arginine metabolism. Biomed. Pharmacother. Biomed. Pharmacother. 171, 116192. doi: 10.1016/j.biopha.2024.116192 38262153

[B12] Chou R. Cantor A. Dana T. Wagner J. Ahmed A. Y. Fu R. . (2022). Statin use for the primary prevention of cardiovascular disease in adults: updated evidence report and systematic review for the US preventive services task force. JAMA 328, 754–771. doi: 10.1001/jama.2022.12138 35997724

[B13] Cooper-DeHoff R. M. Niemi M. Ramsey L. B. Luzum J. A. Tarkiainen E. K. Straka R. J. . (2022). The clinical pharmacogenetics implementation consortium guideline for SLCO1B1, ABCG2, and CYP2C9 genotypes and statin-associated musculoskeletal symptoms. Clin. Pharmacol. Ther. 111, 1007–1021. doi: 10.1002/cpt.2557 35152405 PMC9035072

[B14] Dehnavi S. Sohrabi N. Sadeghi M. Lansberg P. Banach M. Al-Rasadi K. . (2020). Statins and autoimmunity: state-of-the-art. Pharmacol. Ther. 214, 107614. doi: 10.1016/j.pharmthera.2020.107614 32592715

[B15] Elsby R. Hilgendorf C. Fenner K. (2012). Understanding the critical disposition pathways of statins to assess drug-drug interaction risk during drug development: it’s not just about OATP1B1. Clin. Pharmacol. Ther. 92, 584–598. doi: 10.1038/clpt.2012.163 23047648

[B16] Evans L. Rhodes A. Alhazzani W. Antonelli M. Coopersmith C. M. French C. . (2021). Surviving sepsis campaign: international guidelines for management of sepsis and septic shock 2021. Crit. Care Med. 49, e1063–e1143. doi: 10.1097/CCM.0000000000005337 34605781

[B17] Fleischmann C. Scherag A. Adhikari N. K. J. Hartog C. S. Tsaganos T. Schlattmann P. . (2016). Assessment of global incidence and mortality of hospital-treated sepsis. Current estimates and limitations. Am. J. Respir. Crit. Care Med. 193, 259–272. doi: 10.1164/rccm.201504-0781OC 26414292

[B18] Ford I. Murray H. McCowan C. Packard C. J. (2016). Long-term safety and efficacy of lowering low-density lipoprotein cholesterol with statin therapy: 20-year follow-up of west of Scotland coronary prevention study. Circulation 133, 1073–1080. doi: 10.1161/CIRCULATIONAHA.115.019014 26864092 PMC4894764

[B19] Girardis M. David S. Ferrer R. Helms J. Juffermans N. P. Martin-Loeches I. . (2024). Understanding, assessing and treating immune, endothelial and haemostasis dysfunctions in bacterial sepsis. Intensive Care Med. 50, 1580–1592. doi: 10.1007/s00134-024-07586-2 39222142

[B20] Gitomer B. Y. Ostrow A. Wang W. George D. Coleman E. Nowak K. L. . (2025). A randomized controlled trial evaluated the effect of pravastatin on kidney disease outcomes in adult patients with early-stage autosomal dominant polycystic kidney disease. Kidney Int. 109(2), 390–397. doi: 10.1016/j.kint.2025.08.037 41077128

[B21] Habibzadeh F. (2017). Statistical data editing in scientific articles. J. Korean Med. Sci. 32, 1072–1076. doi: 10.3346/jkms.2017.32.7.1072 28581261 PMC5461308

[B22] Hague W. E. Simes J. Kirby A. Keech A. C. White H. D. Hunt D. . (2016). Long-term effectiveness and safety of pravastatin in patients with coronary heart disease: sixteen years of follow-up of the LIPID study. Circulation 133, 1851–1860. doi: 10.1161/CIRCULATIONAHA.115.018580 27016105

[B23] Hirota T. Fujita Y. Ieiri I. (2020). An updated review of pharmacokinetic drug interactions and pharmacogenetics of statins. Expert Opin. Drug Metab. Toxicol. 16, 809–822. doi: 10.1080/17425255.2020.1801634 32729746

[B24] Hodkinson A. Tsimpida D. Kontopantelis E. Rutter M. K. Mamas M. A. Panagioti M. (2022). Comparative effectiveness of statins on non-high density lipoprotein cholesterol in people with diabetes and at risk of cardiovascular disease: systematic review and network meta-analysis. BMJ 376, e067731. doi: 10.1136/bmj-2021-067731 35331984 PMC8943592

[B25] Jain M. K. Ridker P. M. (2005). Anti-inflammatory effects of statins: clinical evidence and basic mechanisms. Nat. Rev. Drug Discov. 4, 977–987. doi: 10.1038/nrd1901 16341063

[B26] Joffre J. Hellman J. Ince C. Ait-Oufella H. (2020). Endothelial responses in sepsis. Am. J. Respir. Crit. Care Med. 202, 361–370. doi: 10.1164/rccm.201910-1911TR 32101446

[B27] Kansal V. Burnham A. J. Kinney B. L. C. Saba N. F. Paulos C. Lesinski G. B. . (2023). Statin drugs enhance responses to immune checkpoint blockade in head and neck cancer models. J. ImmunoTher. Cancer 11, e005940. doi: 10.1136/jitc-2022-005940 36650022 PMC9853267

[B28] Koushki K. Shahbaz S. K. Mashayekhi K. Sadeghi M. Zayeri Z. D. Taba M. Y. . (2021). Anti-inflammatory action of statins in cardiovascular disease: the role of inflammasome and toll-like receptor pathways. Clin. Rev. Allergy Immunol. 60, 175–199. doi: 10.1007/s12016-020-08791-9 32378144 PMC7985098

[B29] Kruger P. Bailey M. Bellomo R. Cooper D. J. Harward M. Higgins A. . (2013). A multicenter randomized trial of atorvastatin therapy in intensive care patients with severe sepsis. Am. J. Respir. Crit. Care Med. 187, 743–750. doi: 10.1164/rccm.201209-1718OC 23348980

[B30] Lee M. G. Lee C.-C. Lai C.-C. Hsu T.-C. Porta L. Lee M. . (2017). Preadmission statin use improves the outcome of less severe sepsis patients - a population-based propensity score matched cohort study. Br. J. Anaesth. 119, 645–654. doi: 10.1093/bja/aex294 29121292

[B31] Lelubre C. Vincent J.-L. (2018). Mechanisms and treatment of organ failure in sepsis. Nat. Rev. Nephrol. 14, 417–427. doi: 10.1038/s41581-018-0005-7 29691495

[B32] Li C. Zhao K. Ren Q. Chen L. Zhang Y. Wang G. . (2025). Statin use during intensive care unit stay is associated with improved clinical outcomes in critically ill patients with sepsis: a cohort study. Front. Immunol. 16, 1537172. doi: 10.3389/fimmu.2025.1537172 40547040 PMC12179067

[B33] Li M. Noordam R. Trompet S. Winter E. M. Jukema J. W. Arbous M. S. . (2024). The impact of statin use on sepsis mortality. J. Clin. Lipidol. 18, e915. doi: 10.1016/j.jacl.2024.07.006 39299824

[B34] Li Y. Tang S. Wang H. Zhu H. Lu Y. Zhang Y. . (2025). A pancreatic cancer organoid biobank links multi-omics signatures to therapeutic response and clinical evaluation of statin combination therapy. Cell Stem Cell 32, 1369–1389.e14. doi: 10.1016/j.stem.2025.07.008 40812300

[B35] Liu D. Huang S.-Y. Sun J.-H. Zhang H.-C. Cai Q.-L. Gao C. . (2022). Sepsis-induced immunosuppression: mechanisms, diagnosis and current treatment options. Mil. Med. Res. 9, 56. doi: 10.1186/s40779-022-00422-y 36209190 PMC9547753

[B36] Liu C. Shen M. Tan W. L. W. Chen I. Y. Liu Y. Yu X. . (2023). Statins improve endothelial function via suppression of epigenetic-driven EndMT. Nat. Cardiovasc. Res. 2, 467–485. doi: 10.1038/s44161-023-00267-1 37693816 PMC10489108

[B37] Lonjon G. Porcher R. Ergina P. Fouet M. Boutron I. (2017). Potential pitfalls of reporting and bias in observational studies with propensity score analysis assessing a surgical procedure: a methodological systematic review. Ann. Surg. 265, 901–909. doi: 10.1097/SLA.0000000000001797 27232253

[B38] Mackinnon A. (2010). The use and reporting of multiple imputation in medical research - a review. J. Intern. Med. 268, 586–593. doi: 10.1111/j.1365-2796.2010.02274.x 20831627

[B39] McAuley D. F. Laffey J. G. O’Kane C. M. Perkins G. D. Mullan B. Trinder T. J. . (2014). Simvastatin in the acute respiratory distress syndrome. N. Engl. J. Med. 371, 1695–1703. doi: 10.1056/NEJMoa1403285 25268516

[B40] Oesterle A. Laufs U. Liao J. K. (2017). Pleiotropic effects of statins on the cardiovascular system. Circ. Res. 120, 229–243. doi: 10.1161/CIRCRESAHA.116.308537 28057795 PMC5467317

[B41] Park C. Jang J.-H. Kim C. Lee Y. Lee E. Yang H.-M. . (2024). Real-world effectiveness of statin therapy in adult asthma. J. Allergy Clin. Immunol. Pract. 12, 399–408.e6. doi: 10.1016/j.jaip.2023.10.029 37866433

[B42] Park J. H. Mortaja M. Son H. G. Zhao X. Sloat L. M. Azin M. . (2024). Statin prevents cancer development in chronic inflammation by blocking interleukin 33 expression. Nat. Commun. 15, 4099. doi: 10.1038/s41467-024-48441-8 38816352 PMC11139893

[B43] Royall D. R. Palmer R. F. Alzheimer’s Disease Neuroimaging Initiative (ADNI) (2024). Statin use moderates APOE’s and CRP’s associations with dementia and is associated with lesser dementia severity in ϵ4 carriers. Alzheimers Dement J. Alzheimers Assoc. 20, 1627–1636. doi: 10.1002/alz.13543 38055626 PMC10984456

[B44] Rudd K. E. Johnson S. C. Agesa K. M. Shackelford K. A. Tsoi D. Kievlan D. R. . (2020). Global, regional, and national sepsis incidence and mortality, 1990-2017: analysis for the global burden of disease study. Lancet Lond. Engl. 395, 200–211. doi: 10.1016/S0140-6736(19)32989-7 31954465 PMC6970225

[B45] Seymour C. W. Liu V. X. Iwashyna T. J. Brunkhorst F. M. Rea T. D. Scherag A. . (2016). Assessment of clinical criteria for sepsis: for the third international consensus definitions for sepsis and septic shock (sepsis-3). JAMA 315, 762–774. doi: 10.1001/jama.2016.0288 26903335 PMC5433435

[B46] Singer M. Deutschman C. S. Seymour C. W. Shankar-Hari M. Annane D. Bauer M. . (2016). The third international consensus definitions for sepsis and septic shock (sepsis-3). JAMA 315, 801–810. doi: 10.1001/jama.2016.0287 26903338 PMC4968574

[B47] Sterne J. A. C. White I. R. Carlin J. B. Spratt M. Royston P. Kenward M. G. . (2009). Multiple imputation for missing data in epidemiological and clinical research: potential and pitfalls. BMJ 338, b2393. doi: 10.1136/bmj.b2393 19564179 PMC2714692

[B48] Tajbakhsh A. Gheibihayat S. M. Askari H. Savardashtaki A. Pirro M. Johnston T. P. . (2022). Statin-regulated phagocytosis and efferocytosis in physiological and pathological conditions. Pharmacol. Ther. 238, 108282. doi: 10.1016/j.pharmthera.2022.108282 36130624

[B49] Terblanche M. Almog Y. Rosenson R. S. Smith T. S. Hackam D. G. (2007). Statins and sepsis: multiple modifications at multiple levels. Lancet Infect. Dis. 7, 358–368. doi: 10.1016/S1473-3099(07)70111-1 17448939

[B50] Toghi C. J. Martins L. Z. Pacheco L. L. Caetano E. S. P. Mattos B. R. Rizzi E. . (2023). Pravastatin prevents increases in activity of metalloproteinase-2 and oxidative stress, and enhances endothelium-derived nitric oxide-dependent vasodilation in gestational hypertension. Antioxid. Basel Switz 12, 939. doi: 10.3390/antiox12040939 37107314 PMC10135677

[B51] Tonelli M. Isles C. Craven T. Tonkin A. Pfeffer M. A. Shepherd J. . (2005). Effect of pravastatin on rate of kidney function loss in people with or at risk for coronary disease. Circulation 112, 171–178. doi: 10.1161/CIRCULATIONAHA.104.517565 15998677

[B52] Tong S. Kaitu’u-Lino T. J. Hastie R. Brownfoot F. Cluver C. Hannan N. (2022). Pravastatin, proton-pump inhibitors, metformin, micronutrients, and biologics: new horizons for the prevention or treatment of preeclampsia. Am. J. Obstet. Gynecol 226, S1157–S1170. doi: 10.1016/j.ajog.2020.09.014 32946849

[B53] van den Hoek H. L. Bos W. J. W. de Boer A. van de Garde E. M. W. (2011). Statins and prevention of infections: systematic review and meta-analysis of data from large randomised placebo controlled trials. BMJ 343, d7281. doi: 10.1136/bmj.d7281 22127443 PMC3226140

[B54] Wan Y.-D. Sun T.-W. Kan Q.-C. Guan F.-X. Zhang S.-G. (2014). Effect of statin therapy on mortality from infection and sepsis: a meta-analysis of randomized and observational studies. Crit. Care Lond. Engl. 18, R71. doi: 10.1186/cc13828 24725598 PMC4056771

[B55] Westphal Filho F. L. Moss Lopes P. R. Menegaz de Almeida A. Sano V. K. T. Tamashiro F. M. Gonçalves O. R. . (2025). Statin use and dementia risk: a systematic review and updated meta-analysis. Alzheimers Dement N. Y. N. 11, e70039. doi: 10.1002/trc2.70039 39822593 PMC11736423

[B56] Yang W. S. Kang H. D. Jung S. K. Lee Y. J. Oh S. H. Kim Y.-J. . (2020). A mortality analysis of septic shock, vasoplegic shock and cryptic shock classified by the third international consensus definitions (sepsis-3). Clin. Respir. J. 14, 857–863. doi: 10.1111/crj.13218 32438528

[B57] Yu D. Liao J. K. (2022). Emerging views of statin pleiotropy and cholesterol lowering. Cardiovasc. Res. 118, 413–423. doi: 10.1093/cvr/cvab032 33533892 PMC8803071

[B58] Zeiser R. (2018). Immune modulatory effects of statins. Immunology 154, 69–75. doi: 10.1111/imm.12902 29392731 PMC5904709

[B59] Zhan X. Huang H. Ye L. Zhu S. Chen J. (2025). The impact of early use of statin in sepsis patients with acute kidney injury: a study based on MIMIC-IV. Front. Pharmacol. 16, 1610450. doi: 10.3389/fphar.2025.1610450 40620675 PMC12226583

[B60] Zhang Y.-Y. Ning B.-T. (2021). Signaling pathways and intervention therapies in sepsis. Signal. Transduct Target Ther. 6, 407. doi: 10.1038/s41392-021-00816-9 34824200 PMC8613465

