# Supplementary material for: Association between pravastatin use and short-term mortality in ICU patients with sepsis: a retrospective propensity score-matched cohort study
Source: Front Cell Infect Microbiol. 2026 Jul 3;16:1814760. doi: 10.3389/fcimb.2026.1814760 (PMC13375620; doi:10.3389/fcimb.2026.1814760)
Supplement: Supplementary file 1 [file Table1.docx]

**Table S1.** Missing Data Summary for Study Variables

| **Variable** | **Missing, n** | **Missing, %** |
| --- | --- | --- |
| Age | 0 | 0.00 |
| Gender | 0 | 0.00 |
| Race | 2365 | 15.03 |
| Height | 6286 | 39.96 |
| Weight | 133 | 0.85 |
| Charlson Comorbidity Index | 0 | 0.00 |
| SOFA Score | 0 | 0.00 |
| Heart Rate | 0 | 0.00 |
| Systolic Blood Pressure | 160 | 1.02 |
| Diastolic Blood Pressure | 160 | 1.02 |
| Mean Arterial Pressure | 160 | 1.02 |
| Respiratory Rate | 5 | 0.03 |
| SpO₂ | 6 | 0.04 |
| Temperature | 380 | 2.42 |
| White Blood Cell Count | 91 | 0.58 |
| Red Blood Cell Count | 81 | 0.51 |
| Platelet Count | 108 | 0.69 |
| Hemoglobin | 84 | 0.53 |
| Neutrophil Count | 10694 | 67.98 |
| Lymphocyte Count | 10694 | 67.98 |
| Albumin | 5268 | 33.49 |
| Alanine Aminotransferase | 3796 | 24.13 |
| Aspartate Aminotransferase | 3706 | 23.56 |
| Direct Bilirubin | 12839 | 81.61 |
| Total Bilirubin | 3808 | 24.21 |
| Total Serum Calcium | 235 | 1.49 |
| Chloride | 23 | 0.15 |
| Sodium | 18 | 0.11 |
| Potassium | 31 | 0.20 |
| Globulin | 15205 | 96.65 |
| Total protein | 14519 | 92.29 |
| Creatine Kinase | 9020 | 57.34 |
| Creatine Kinase-MB | 10043 | 63.84 |
| Blood Urea Nitrogen | 22 | 0.14 |
| Creatinine | 20 | 0.13 |
| Uric Acid | 14712 | 93.52 |
| D-Dimer | 15318 | 97.37 |
| Fibrinogen | 9425 | 59.91 |
| Glucose | 18 | 0.11 |
| HbA1c | 14109 | 89.68 |
| Anion Gap | 79 | 0.50 |
| Lactate | 2692 | 17.11 |
| PaO₂/FiO₂ Ratio | 6464 | 41.09 |
| PaCO₂ | 2765 | 17.58 |
| pH | 2484 | 15.79 |
| PaO₂ | 2752 | 17.49 |
| INR | 858 | 5.45 |
| Total Cholesterol | 14456 | 91.89 |
| High-Density Lipoprotein | 14609 | 92.86 |
| Low-Density Lipoprotein | 14677 | 93.29 |
| Triglycerides | 11988 | 76.20 |
| Troponin T | 11590 | 73.67 |
| Left Ventricular Ejection Fraction | 12647 | 80.39 |
| NT-proBNP | 14249 | 90.57 |
| CRP | 15203 | 96.64 |

**Figure S1.** Propensity Score Distribution Before and After Matching


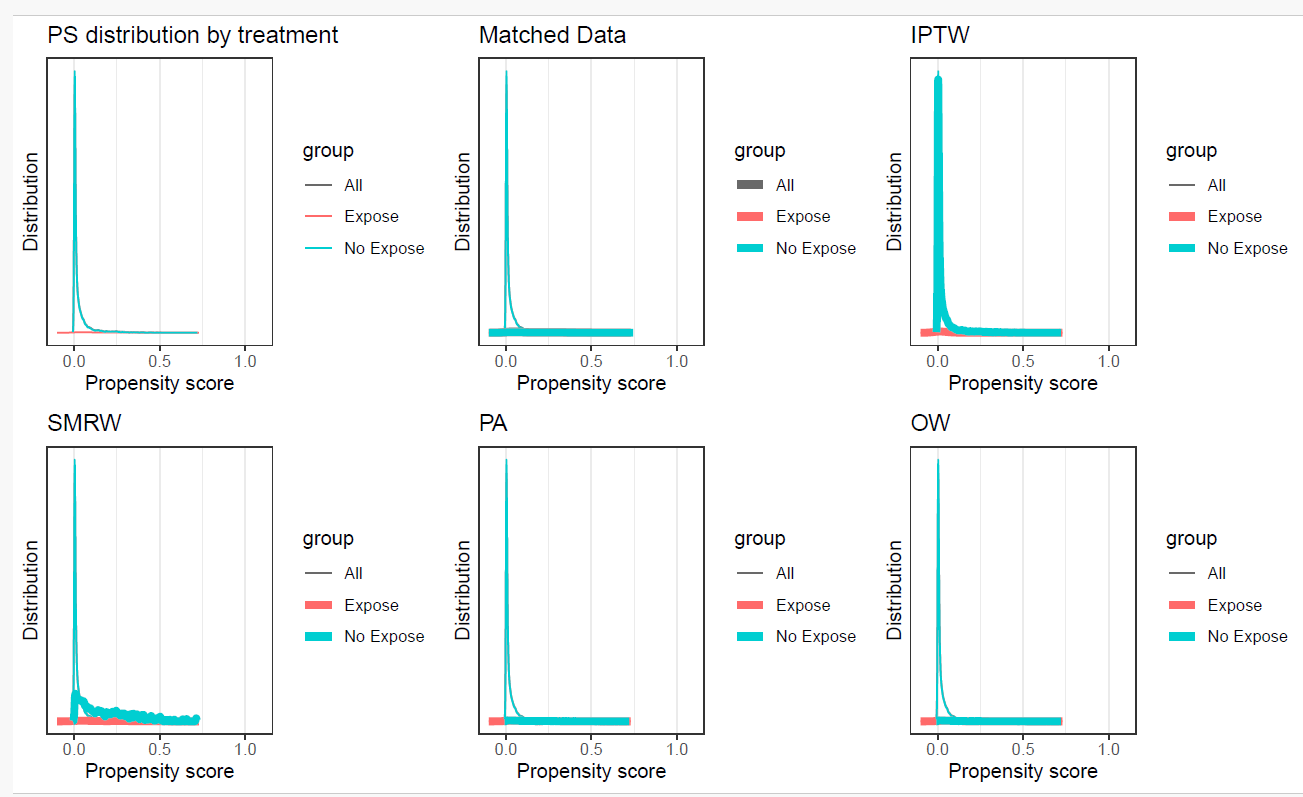


**Figure S2.** Covariate Balance Assessment (Love Plot)


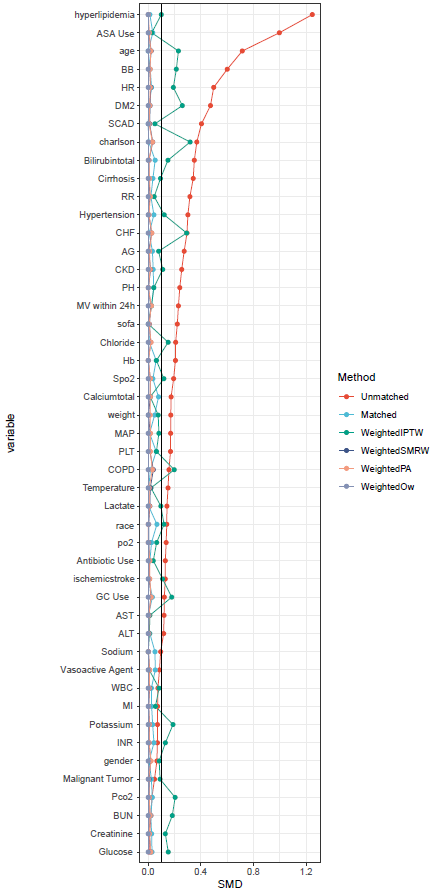


Table S2. Univariate Cox Regression Analysis for 28-Day Mortality Before PSM

| Variable | Hazard ratio (95% CI) | P value |
| --- | --- | --- |
| Pravastatin use: 1 vs 0 | 0.47 (0.37-0.60) | <0.001 |
| Hyperlipidemia: 1 vs 0 | 1.18 (1.10-1.27) | <0.001 |
| SCAD: 1 vs 0 | 1.63 (1.45-1.83) | <0.001 |
| Ischemic stroke: 1 vs 0 | 1.63 (1.48-1.81) | <0.001 |
| Age, per 1 year | 1.03 (1.02-1.03) | <0.001 |
| Race: ref. = White |  | <0.001 |
| Other | 0.93 (0.85-1.01) | 0.088 |
| Unknown | 1.63 (1.50-1.77) | <0.001 |
| Female sex: 2 vs 1 | 1.07 (1.00-1.14) | 0.059 |
| Weight, per 10 kg | 0.946 (0.932-0.960) | <0.001 |
| WBC, per 10 x10^9/L | 1.062 (1.047-1.078) | <0.001 |
| Platelet count, per 100 x10^9/L | 0.951 (0.923-0.980) | <0.001 |
| Hemoglobin, per 1 g/dL | 0.95 (0.93-0.96) | <0.001 |
| Sodium, per 1 mmol/L | 0.9947 (0.989-1.0005) | 0.071 |
| Potassium, per 1 mmol/L | 1.26 (1.21-1.30) | <0.001 |
| Total serum calcium, per 1 mg/dL | 1.04 (1.00-1.08) | 0.039 |
| Chloride, per 1 mmol/L | 0.97 (0.97-0.98) | <0.001 |
| Glucose, per 10 mg/dL | 1.014 (1.010-1.017) | <0.001 |
| Anion gap, per 1 mmol/L | 1.08 (1.08-1.09) | <0.001 |
| pH, per 1 unit | 0.07 (0.05-0.09) | <0.001 |
| PaCO2, per 10 mmHg | 1.043 (1.016-1.069) | 0.002 |
| PaO2, per 10 mmHg | 0.927 (0.920-0.935) | <0.001 |
| Lactate, per 1 mmol/L | 1.16 (1.15-1.17) | <0.001 |
| INR, per 1 unit | 1.19 (1.17-1.21) | <0.001 |
| Total bilirubin, per 1 mg/dL | 1.04 (1.04-1.05) | <0.001 |
| ALT, per 100 U/L | 1.010 (1.000-1.010) | 0.003 |
| AST, per 100 U/L | 1.010 (1.000-1.010) | <0.001 |
| BUN, per 1 mg/dL | 1.01 (1.01-1.02) | <0.001 |
| Creatinine, per 1 mg/dL | 1.09 (1.08-1.10) | <0.001 |
| Heart rate, per 10 bpm | 1.046 (1.038-1.053) | <0.001 |
| MAP, per 10 mmHg | 0.978 (0.961-0.996) | 0.015 |
| Respiratory rate, per 1 breath/min | 1.03 (1.03-1.04) | <0.001 |
| SpO2, per 1 percentage point | 0.97 (0.96-0.97) | <0.001 |
| Temperature, per 1 deg C | 0.79 (0.76-0.83) | <0.001 |
| Vasoactive agent use within 24 h after ICU admission: 1 vs 0 | 1.84 (1.72-1.97) | <0.001 |
| MV within 24 h after ICU admission: 1 vs 0 | 1.16 (1.08-1.24) | <0.001 |
| SOFA score, per 1 point | 1.16 (1.15-1.17) | <0.001 |
| Charlson Comorbidity Index, per 1 point | 1.19 (1.17-1.20) | <0.001 |
| Hypertension: 1 vs 0 | 0.97 (0.91-1.04) | 0.429 |
| T2DM: 1 vs 0 | 1.20 (1.11-1.29) | <0.001 |
| CHF: 1 vs 0 | 1.48 (1.38-1.59) | <0.001 |
| MI: 1 vs 0 | 2.47 (2.16-2.81) | <0.001 |
| Malignancy: 1 vs 0 | 1.80 (1.66-1.95) | <0.001 |
| CKD: 1 vs 0 | 1.54 (1.42-1.67) | <0.001 |
| Cirrhosis: 1 vs 0 | 1.61 (1.48-1.75) | <0.001 |
| COPD: 1 vs 0 | 1.55 (1.39-1.73) | <0.001 |
| GC use: 1 vs 0 | 1.19 (1.11-1.28) | <0.001 |
| ASA use: 1 vs 0 | 0.57 (0.52-0.62) | <0.001 |
| BB use: 1 vs 0 | 0.68 (0.63-0.73) | <0.001 |
| Antibiotic use within 24 h after ICU admission: 1 vs 0 | 0.96 (0.87-1.06) | 0.407 |

Abbreviations: AG, Anion Gap; ALT, Alanine Aminotransferase; ASA, Acetylsalicylic Acid; AST, Aspartate Aminotransferase; BB, Beta-adrenergic Blocking Agent; BUN, Blood Urea Nitrogen; CHF, Congestive Heart Failure; CKD, Chronic Kidney Disease; COPD, Chronic Obstructive Pulmonary Disease; GC, Glucocorticoid; Hb, Hemoglobin; INR, International Normalized Ratio; MAP, Mean Arterial Pressure; MI, Myocardial Infarction; MV, Mechanical Ventilation; PaCO₂, Arterial Partial Pressure of Carbon Dioxide; PaO₂, Arterial Partial Pressure of Oxygen; PLT, Platelet; SCAD, Stable Coronary Artery Disease; SOFA, Sequential Organ Failure Assessment; SpO₂, Peripheral Oxygen Saturation; T2DM, Type 2 Diabetes Mellitus; WBC, White Blood Cell

Table S3. Univariate Cox Regression Analysis for 28-Day Mortality After PSM

| Variable | Hazard ratio (95% CI) | P value |
| --- | --- | --- |
| Pravastatin use: 1 vs 0 | 0.66 (0.48-0.90) | 0.009 |
| Age, per 1 year | 1.06 (1.05-1.08) | <0.001 |
| Female sex: 2 vs 1 | 0.92 (0.67-1.26) | 0.605 |
| Weight, per 10 kg | 0.914 (0.851-0.980) | 0.018 |
| Race: ref. = White |  | 0.001 |
| Other | 0.57 (0.35-0.93) | 0.024 |
| Unknown | 1.64 (1.09-2.45) | 0.017 |
| Heart rate, per 10 bpm | 1.219 (1.105-1.344) | <0.001 |
| MAP, per 10 mmHg | 1.068 (0.980-1.172) | 0.143 |
| Respiratory rate, per 1 breath/min | 1.04 (1.03-1.06) | <0.001 |
| SpO2, per 1 percentage point | 0.95 (0.92-0.99) | 0.006 |
| Temperature, per 1 deg C | 0.90 (0.69-1.17) | 0.443 |
| WBC, per 10 x10^9/L | 1.219 (1.105-1.480) | <0.001 |
| Platelet count, per 100 x10^9/L | 1.209 (1.020-1.418) | 0.024 |
| Hemoglobin, per 1 g/dL | 0.9947 (0.921-1.075) | 0.893 |
| Sodium, per 1 mmol/L | 1.02 (0.99-1.06) | 0.190 |
| Potassium, per 1 mmol/L | 1.14 (0.92-1.42) | 0.221 |
| Total serum calcium, per 1 mg/dL | 1.07 (0.88-1.30) | 0.513 |
| Chloride, per 1 mmol/L | 0.96 (0.94-0.98) | <0.001 |
| Glucose, per 10 mg/dL | 1.027 (1.010-1.041) | <0.001 |
| Anion gap, per 1 mmol/L | 1.11 (1.08-1.15) | <0.001 |
| pH, per 1 unit | 0.02 (0.01-0.07) | <0.001 |
| PaCO2, per 10 mmHg | 1.105 (1.000-1.344) | 0.037 |
| PaO2, per 10 mmHg | 0.908 (0.869-0.951) | <0.001 |
| Lactate, per 1 mmol/L | 1.08 (0.98-1.19) | 0.125 |
| INR, per 1 unit | 1.23 (1.03-1.47) | 0.021 |
| Total bilirubin, per 1 mg/dL | 1.03 (0.89-1.18) | 0.695 |
| ALT, per 100 U/L | 1.030 (1.000-1.051) | 0.034 |
| AST, per 100 U/L | 1.020 (1.010-1.030) | 0.006 |
| BUN, per 1 mg/dL | 1.02 (1.01-1.02) | <0.001 |
| Creatinine, per 1 mg/dL | 1.13 (1.06-1.20) | <0.001 |
| Hypertension: 1 vs 0 | 0.65 (0.47-0.89) | 0.007 |
| T2DM: 1 vs 0 | 1.22 (0.90-1.67) | 0.205 |
| CHF: 1 vs 0 | 1.85 (1.36-2.53) | <0.001 |
| MI: 1 vs 0 | 3.00 (1.81-4.96) | <0.001 |
| Malignancy: 1 vs 0 | 1.79 (1.25-2.56) | 0.001 |
| CKD: 1 vs 0 | 1.52 (1.09-2.12) | 0.014 |
| Cirrhosis: 1 vs 0 | 0.88 (0.39-2.00) | 0.769 |
| COPD: 1 vs 0 | 2.01 (1.37-2.97) | <0.001 |
| Hyperlipidemia: 1 vs 0 | 0.89 (0.62-1.28) | 0.532 |
| SCAD: 1 vs 0 | 1.20 (0.82-1.76) | 0.347 |
| Ischemic stroke: 1 vs 0 | 1.55 (1.02-2.34) | 0.039 |
| Vasoactive agent use within 24 h after ICU admission: 1 vs 0 | 1.66 (1.20-2.30) | 0.002 |
| MV within 24 h after ICU admission: 1 vs 0 | 0.80 (0.59-1.10) | 0.171 |
| GC use: 1 vs 0 | 1.58 (1.11-2.24) | 0.011 |
| ASA use: 1 vs 0 | 0.57 (0.42-0.78) | <0.001 |
| BB use: 1 vs 0 | 0.58 (0.42-0.79) | <0.001 |
| Antibiotic use within 24 h after ICU admission: 1 vs 0 | 0.69 (0.43-1.13) | 0.146 |
| SOFA score, per 1 point | 1.19 (1.13-1.25) | <0.001 |
| Charlson Comorbidity Index, per 1 point | 1.23 (1.17-1.28) | <0.001 |

Abbreviations: AG, Anion Gap; ALT, Alanine Aminotransferase; ASA, Acetylsalicylic Acid; AST, Aspartate Aminotransferase; BB, Beta-adrenergic Blocking Agent; BUN, Blood Urea Nitrogen; CHF, Congestive Heart Failure; CKD, Chronic Kidney Disease; COPD, Chronic Obstructive Pulmonary Disease; GC, Glucocorticoid; Hb, Hemoglobin; INR, International Normalized Ratio; MAP, Mean Arterial Pressure; MI, Myocardial Infarction; MV, Mechanical Ventilation; PaCO₂, Arterial Partial Pressure of Carbon Dioxide; PaO₂, Arterial Partial Pressure of Oxygen; PLT, Platelet; SCAD, Stable Coronary Artery Disease; SOFA, Sequential Organ Failure Assessment; SpO₂, Peripheral Oxygen Saturation; T2DM, Type 2 Diabetes Mellitus; WBC, White Blood Cell

Table S4. Multicollinearity diagnostics for the included covariates.

| Variables | Tolerance | VIF |
| --- | --- | --- |
| Ischemic stroke | 0.31834 | 3.141298 |
| ALT | 0.108121 | 9.248911 |
| CHF | 0.125125 | 7.992037 |
| Hypertension | 0.204804 | 4.882707 |
| age | 0.122251 | 8.179875 |
| MI | 0.201897 | 4.953028 |
| Race | 0.283978 | 3.521401 |
| Gender | 0.23563 | 4.243948 |
| Weight | 0.115043 | 8.6924 |
| WBC | 0.282259 | 3.542851 |
| BB Use | 0.341877 | 2.925027 |
| PLT | 0.349809 | 2.8587 |
| Chloride | 0.188499 | 5.305074 |
| Glucose | 0.276065 | 3.622335 |
| pH | 0.151775 | 6.588686 |
| PaO₂ | 0.37024 | 2.700947 |
| INR | 0.115185 | 8.681701 |
| BUN | 0.264092 | 3.786557 |
| Creatinine | 0.122466 | 8.165557 |
| Heart Rate | 0.221244 | 4.519905 |
| MAP | 0.270491 | 3.696985 |
| Respiratory Rate | 0.319633 | 3.128589 |
| Spo2 | 0.401728 | 2.489248 |
| Temperature | 0.355836 | 2.810281 |
| Vasoactive Agent Use within 24h after ICU admission | 0.263077 | 3.801163 |
| COPD | 0.604273 | 1.654882 |
| SOFA score | 0.124513 | 8.031315 |
| PaCO₂ | 0.197115 | 5.073176 |
| GC Use | 0.395791 | 2.526588 |
| ASA Use | 0.058501 | 17.09364 |
| Malignancy | 0.083846 | 11.92657 |
| AG | 0.060849 | 16.43419 |
| CKD | 0.034175 | 29.26125 |
| Charlson Comorbidity Index | 0.000122 | 8229.299 |
| AST | 0.036644 | 27.28899 |

VIF, variance inflation factor.

**Table S5. Sensitivity Analyses for the Association Between Pravastatin Use and 28-Day Mortality in the Matched Cohort**

| Outcome | RR (95%CI) | E-value Point | E-value Lower 95%CI |
| --- | --- | --- | --- |
| 28-day mortality | 1.45 (1.05–2.00) | 2.26 | 1.29 |
